# Resistance Trend Estimation Using Regression Analysis to Enhance Antimicrobial Surveillance: A Multi-Centre Study in London 2009–2016

**DOI:** 10.3390/antibiotics10101267

**Published:** 2021-10-18

**Authors:** Bernard Hernandez, Pau Herrero-Viñas, Timothy M. Rawson, Luke S. P. Moore, Alison H. Holmes, Pantelis Georgiou

**Affiliations:** 1 Centre for Bio-Inspired Technology, Department of Electrical and Electronic Engineering, Imperial College London, London SW7 2AZ, UK; p.herrero-vinias@imperial.ac.uk (P.H.-V.); pantelis@imperial.ac.uk (P.G.); 2 Centre for Antimicrobial Optimisation (CAMO), Imperial College London, London W12 0NN, UK; timothy.rawson07@imperial.ac.uk (T.M.R.); alison.holmes@imperial.ac.uk (A.H.H.); 3 National Institute for Health Research Health Protection Research Unit in Healthcare Associated Infections and Antimicrobial Resistance, Imperial College London, London W12 0NN, UK; 4 Chelsea and Westminster NHS Foundation Trust, London SW10 9NH, UK; l.moore@imperial.ac.uk

**Keywords:** antimicrobial resistance, resistance rate, resistance trend, antimicrobial surveillance, regression analysis, time series analysis, escherichia coli, staphylococcus aureus

## Abstract

In the last years, there has been an increase of antimicrobial resistance rates around the world with the misuse and overuse of antimicrobials as one of the main leading drivers. In response to this threat, a variety of initiatives have arisen to promote the efficient use of antimicrobials. These initiatives rely on antimicrobial surveillance systems to promote appropriate prescription practices and are provided by national or global health care institutions with limited consideration of the variations within hospitals. As a consequence, physicians’ adherence to these generic guidelines is still limited. To fill this gap, this work presents an automated approach to performing local antimicrobial surveillance from microbiology data. Moreover, in addition to the commonly reported resistance rates, this work estimates secular resistance trends through regression analysis to provide a single value that effectively communicates the resistance trend to a wider audience. The methods considered for trend estimation were ordinary least squares regression, weighted least squares regression with weights inversely proportional to the number of microbiology records available and autoregressive integrated moving average. Among these, weighted least squares regression was found to be the most robust against changes in the granularity of the time series and presented the best performance. To validate the results, three case studies have been thoroughly compared with the existing literature: (i) *Escherichia coli* in urine cultures; (ii) *Escherichia coli* in blood cultures; and (iii) *Staphylococcus aureus* in wound cultures. The benefits of providing local rather than general antimicrobial surveillance data of a higher quality is two fold. Firstly, it has the potential to stimulate engagement among physicians to strengthen their knowledge and awareness on antimicrobial resistance which might encourage prescribers to change their prescription habits more willingly. Moreover, it provides fundamental knowledge to the wide range of stakeholders to revise and potentially tailor existing guidelines to the specific needs of each hospital.

## 1. Introduction

The growing threat of antimicrobial resistance (AMR) is a leading patient health and safety issue, with estimates that AMR will be responsible for more than 10 million deaths by 2050 [1]. The development of resistance in pathogens is another manifestation of the Darwinian theory of biological evolution [2] and is accelerated by circumstances where selective pressure is exerted. As a result, over the last years, research has focused on identifying the factors contributing to AMR [1,3,4]. The alarming outcomes have motivated the emergence of different strategies and guidelines to analyse, present and ultimately combat antimicrobial resistance. At a national level, Public Health England implemented the English surveillance program for antimicrobial utilisation and resistance (ESPAUR) which provides annual reports as a benchmark for determining appropriate local action [5]. At an international level, the European Centre for Disease Prevention and Control through the European antimicrobial resistance surveillance network (EARS-Net) has created the largest publicly funded system for antimicrobial surveillance in Europe [6]. Furthermore, the World Health Organization has recently implemented the global antimicrobial resistance surveillance system (GLASS) [7] to strengthen the evidence base on AMR and inform decision-making. Unfortunately, despite of these initiatives, homogeneity of antimicrobial policies still produce different AMR outcomes [8].

The misuse and overuse of antimicrobials in humans has been identified as a major driver of AMR [3,9]. Whilst reasons for the misuse and overuse of antimicrobials are complex, a number of factors have been described. At the individual level, physicians often prioritise the management of the patient being treated, paying little regard to the long-term consequences of overusing antimicrobials [10]. Moreover, the majority of antimicrobial prescribing is performed by individuals who are not experts in infection management and may have limited understanding of antimicrobials and AMR [3,11,12,13,14]. To address the challenges posed by AMR, the importance of behaviour change interventions to improve the long-term use of antimicrobials in infection management has been recognised [15,16,17]. Thus, there is potential to improve the prescription behaviour of physicians through the implementation of effective communication strategies to present local rather than general AMR surveillance data.

### 1.1. The Need of Local AMR Surveillance

The guidelines on empirical antibiotic use often disregard local resistance patterns in their recommendations [18]. However, with increasing electronic recording of data, there is a growing interest in the potential secondary use of microbiological laboratory data to provide the necessary information to support antimicrobial stewardship programs (AMS) [19] which are crucial to guide health care organizations designing evidence-based policies to combat AMR [20,21]. Local susceptibility reporting and surveillance has been shown to be determinant to inform empiric antimicrobial therapy selection [22,23,24]. For example, a general hospital in mid-Norway reported lower antimicrobial resistance rates than the rest of countries outside Scandinavia within the blood stream infection cohort [25]. As a consequence, appropriate empiric antibiotic therapy was achieved to a larger extent by tailoring existing guidelines according to the local resistance patterns previously identified.

#### 1.1.1. Measuring Resistance from Susceptibility Data

The most widespread resistance measurement is denoted as Single Antimicrobial Resistance Index (SARI) and evaluates the proportion (or percentage) of hosts harbouring resistant pathogens within a certain population. In scenarios where a pathogen exhibits resistance to numerous antimicrobials, the Multiple Antimicrobial Resistance Index (MARI) evaluates the ratio of antimicrobials to which a pathogen is resistant [26]. These metrics inform clinicians on overall antimicrobial resistance levels; however, they overlook information such as resistance tendency or seasonality. For such purpose, the previously explained indexes are computed on consecutive and independent time intervals to produce resistance time series signals. Unfortunately, these are predominantly analysed by means of visual graphs and described vaguely with expressions such as ‘slightly increased’, ‘decreased’ or ‘remained constant’. The exploitation of computational algorithms to automate the handling and interpretation of large microbiology datasets is still limited [27,28].

#### 1.1.2. Evaluating Tendency on Time Series Signals

Time series analysis comprises statistical methods to extract meaningful statistics and characteristics from time series data which is commonly split into four main components: trend, cyclical fluctuation, seasonal variation and residual effect. The trend represents tendencies and regularities in the time series and it is crucial in domains such as stock market, meteorology or biology. The methods suitable for detection and estimation of trends in a particular domain are determined by: (i) the definition of trend; (ii) the model used for the trend; (iii) the characteristics of the data; and (iv) the application objectives. Linear regression is perhaps the most popular method to estimate trends when the trend is defined as the growth rate of a population. In linear regression, the trend is expressed as a linear function of time where ordinary least squares regression has been widely used due to its simplicity to understand [29]. However, it is highly affected by outliers, non-linearity, correlation among variables and heteroskedasticity [30]. To overcome these flaws, there are alternatives such as weighted least squares regression and robust regression. On the other side, autoregressive integrated moving average (ARIMA) is widely applied in time series analysis and has proven to be robust for short term forecasting based on previous observations [31,32]. A number of variations on the ARIMA model have been developed to consider seasonal variations (SARIMA) or handle multiple time series (VARIMA).

In this study, we compare the robustness of three different regression analysis methods to quantify secular resistance trends. Moreover, the results obtained are compared with those reported in the existing literature for three different case studies: (i) *Escherichia coli* in urine cultures; (ii) *Escherichia coli* in blood cultures; and (iii) *Staphylococcus aureus* in wound cultures.

## 2. Materials and Methods

**Figure 1:**
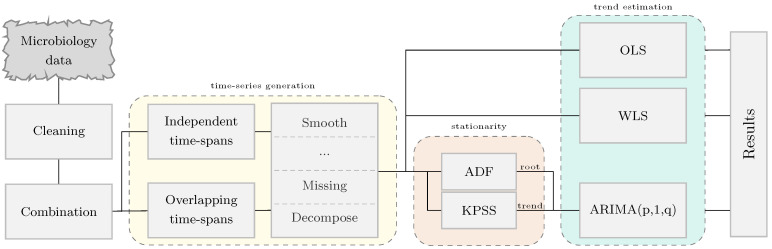
High-level methodology diagram. It is composed by three main sections: time series generation (yellow), stationarity analysis (orange) and trend estimation (green). The stationarity analysis was performed using the Augmented Dickey-Fuller (ADF) and the Kwiatkowski–Phillips–Schmidt–Shin (KPSS) tests to identify root-stationary and trend-stationary time series signals. The regression analysis methods considered were ordinary least squares (OLS), weighted least squares (WLS) and autoregressive integrated moving average (ARIMA).

The methodology implemented to estimate secular trends in AMR from susceptibility data is described in Figure 1. Firstly, the microbiology data was divided into combinations, which are defined by the sample type and a pair pathogen-antimicrobial. For each combination, the resistance time series signal was generated using either independent or overlapping time intervals. The time series was linearly interpolated to fill sporadic missing values. No additional filtering was applied. An analysis of stationarity around a trend was carried out to identify those combinations fulfilling the requirements posed by ARIMA. To conclude, regression analysis was applied to quantify the tendency of the time series.

### 2.1. Microbiology Data

This study was conducted with data from the Imperial College Healthcare NHS Trust, which comprises three separate hospitals. Data contained more than 3.5 million susceptibility tests for over 300,000 isolates corresponding to approximately 200,000 individuals. Laboratory operating procedures followed national standards for microbiological investigation [33]; isolates were identified using API^®^ (bioMèrieux) from 2009 to 2011 and by MALDI-TOF spectroscopy (Biotyper^®^, Brunker) from 2011 to 2015. Susceptibilities were determined by disc diffusion using BSAC criteria [34]. Duplicated or incomplete entries caused by either human (e.g., reporting same results twice accidentally) or software errors were eliminated. In addition, results were de-duplicated to discard identical organisms repeatedly isolated from a patient during the same hospital admission.

### 2.2. Attributes in a Susceptibility Test Record

Susceptibility test records are composed by laboratory number, patient number, date, sample type or culture (e.g., blood or urine), pathogen, antimicrobial, reported status and outcome (resistant, sensitive or intermediate). These were grouped for each sample type by pairs (pathogen, antimicrobial) since it is widely accepted by clinicians as detailed in the UK five year strategy in AMR [20].

### 2.3. Generation of Resistance Time Series Signals

The Single Antimicrobial Resistance Index (SARI) is stated in Equation (1) where R, I and S represent the number of susceptibility tests with resistant, intermediate and susceptible outcomes respectively. It provides a value within the range [0,1] where values close to one indicate high resistance.

(1) $$SARI=\frac{R+I}{R+I+S}.$$

To study the temporal evolution of AMR, it is necessary to generate a resistance time series from the susceptibility test data. This is often achieved by computing the resistance index on consecutive partitions of the data (see Table 1). The traditional strategy of dealing with partitions considers independent time intervals (see yearly, monthly or weekly time series). Unfortunately, this strategy forces to trade-off between granularity (level of detail) and accuracy. The overlapping time intervals strategy drops such dependence by defining a window of fixed size which is moved across time. The length of the window is denoted as period and the time step as shift. For instance, three time series obtained using the overlapping time intervals strategy with a monthly shift (1M) and window lengths of 12, 6 and 3 have been presented for the sake of clarity (see 1M12, 1M6 and 1M3). The notation to define the time series generation methodology (shift_period_) is described with various examples in Table 1. For instance, 7D_4_ defines a time series with weekly resistance indexes (7D) calculated using the microbiology records available for the previous four weeks (4x7D). It is important to note that some notations are equivalent representations of the same susceptibility data at different granularity, hence their slopes are comparable. As an example, the trend estimated for 1M_1_ should be approximately thirty times the trend estimated for 1D_30_.

### 2.4. Regression Analysis for Trend Estimation

The linear model (see Equation (2)) has been selected to quantify resistance tendency for several reasons: (i) the development of resistance in pathogens is an evolutionary response hence large variations in short periods (e.g., consecutive days or months) are not expected; (ii) the slope parameter can be directly translated to change over time increasing its practicability; and (iii) the offset parameter is highly related with the overall resistance. Hence, the response variable in regression analysis (resistance index) is described by the explanatory variable (time). The slope (*m*) ranges within the interval [−1,1] where sign and absolute value capture direction and rate of change respectively. The unit of the slope is represented by $\Delta_{y}/\Delta_{x}$. It has been denoted as Single Antimicrobial Resistance Trend (SART).

(2) $$y=mx+n\;where\;m=\frac{y_{t+1}-y_{t}}{x_{t+1}-x_{t}}.$$

#### 2.4.1. Least Squares Regression

The optimization problem in ordinary least squares (OLS) regression minimizes the least square errors to find the best fitting model as described in Equation (3). These errors ($\epsilon_{i}$) are often called residuals and represent the differences between observed (*y*) and estimated ($y^{\prime }$) variables. Ordinary least squares assumes identical weights ($w_{i}$) and independently distributed residuals with a normal distribution.

(3) $$\min_{m,n}\sum_{i=1}^{T}w_{i}^{2}\epsilon_{i}^{2}\;where\;\epsilon_{i}=y_{i}-y_{i}^{\prime }=yi-\left(mx_{i}+n\right).$$

It is frequently observed that some residuals might have higher variance than others, meaning that those observations are effectively less certain. To contemplate such variability, weighted linear squares (WLS) regression (see Equation (3)) applies a weighting function to the residuals. In this paper, the confidence of the computed resistance index (observed variable) relies on the number of susceptibility test records manipulated. Hence, the sigmoid function has been used to define weights proportional to the population size.

#### 2.4.2. Autoregressive Integrated Moving Average

An autoregressive integrated moving average (ARIMA) model is a generalization of an autoregressive moving average (ARMA) model which can be also applied in scenarios where data show evidence of non-stationarity. The autoregressive (AR) part expresses the variable of interest (resistance index) as a function of past values of the variable. The moving average (MA) indicates that the regression error is a linear combination of error terms which occurred contemporaneously and at various times in the past. An ARIMA(p,d,q) model is defined as shown in Equation (4), where p is the number of autoregressive terms, d is the number of differences needed for stationarity, q is the number of lagged forecast errors, and ϕ and θ are the coefficients of the model.

(4) $$y_{t}^{\prime }=\mu +\sum_{i=1}^{p}\phi_{i}y_{t-i}-\sum_{j=1}^{q}\theta_{j}y_{t-j}.$$

The interpretation of the parameter μ depends on the ARIMA model used for the fitting. In order to estimate the linear trend, it was interesting to consider exclusively MA models so that the expected value of μ was the mean of the one-time differenced series; that is, the slope coefficient of the un-differenced series. The Bayesian information criterion (BIC) was used to select the best ARIMA(0,1,q) model, being the one with the lowest BIC the preferred.

### 2.5. Statistical Analysis

#### 2.5.1. Trend and Stationarity in Time Series

An analysis of stationarity around a trend was carried out to identify time series satisfying the assumptions posed by ARIMA. The augmented Dickey–Fuller test (ADF) was used to determine the presence of a unit root. When the other roots of the characteristic function lie inside the unit circle the first difference of the process is stationary. Due to this property, these are also called difference-stationary processes. Since the absence of unit root is not a proof of non-stationarity, the Kwiatkowski–Phillips–Schmidt–Shin (KPSS) test was used to identify the existence of an underlying trend which can also be removed to obtain a stationary process. These are called trend-stationary processes. In both unit-root and trend-stationary processes, the mean can increase or decrease over time; however, in the presence of a shock, trend-stationary processes revert to this mean tendency in the long run (deterministic trend) while unit-root processes have a permanent impact (stochastic trend). The significance level of the tests was set to 0.05.

#### 2.5.2. Statistical Significance among Regression Methods

The statistical significance of the differences among the regression methods was determined using the non parametric test Wilcoxon–Mann–Whitney (also denoted Mann–Whitney U) where the significance level was set to 0.05.

#### 2.5.3. Pearson Correlation Coefficient

It measures the linear correlation between two variables with a value within the range [−1,1]. Coefficient values of −1, 0 and 1 indicate total negative linear correlation, no linear correlation and total positive correlation respectively. In this study, the coefficient is used to assess whether or not there is a linear correlation between the number of observations (susceptibility test records) and the computed resistance index.

### 2.6. Software

The Python programming language was used in this research. The libraries used for time series analysis and data handling were Statsmodels [35] and Pandas [36] respectively. Additionally, Matplotlib [37] and Seaborn [38] were used for data visualization.

## 3. Results

### 3.1. Analysis of the Robustness of the Methods

**Figure 2:**
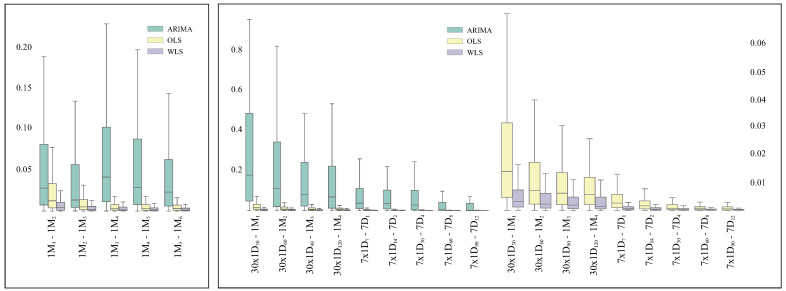
Distribution of paired SART distances. Comparison of ordinary least squares (OLS), weighted least squares (WLS) and autoregressive integrated moving average (ARIMA) in the following scenarios: consecutive periods (**left**) and equivalent granularities (**right**). An additional graph including exclusively OLS and WLS has been added on the latter to facilitate their comparison. The x-axis represents couplets of configurations to generate resistance time series under comparison.

The process to generate a resistance time series signal from susceptibility data is defined by two parameters: shift and period. This section compares the robustness of three regression analysis methods (OLS, WLS and ARIMA) to quantify secular trends for resistance time series generated using different parameter configurations. For such purpose, the absolute difference between paired trends (SART distances) has been computed. The distribution of such distances is shown in Figure 2 for consecutive periods (left) and various granularities (right). Lower values indicate higher consistency in the estimation of trends.

#### 3.1.1. Consistency on Consecutive Time Spans

The length of the period determines the amount of susceptibility test records accounted to compute the resistance index. Lengthy periods provide smoother time series which are better approximated by the linear model, especially when overlapping time periods are considered. As a consequence, the SART distances decrease as shown by the median of the distributions (see left graph in Figure 2). This behaviour is consistent in OLS and WLS. However, it is worth highlighting the irregularities shown by ARIMA. The median and quartiles of the distributions indicate that WLS produces the most stable results and it is followed closely by OLS. Nonetheless, there is a considerable gap between these two methods and ARIMA. All the distances estimated by WLS were significantly smaller (*p* < 0.001) than those obtained using OLS and ARIMA.

#### 3.1.2. Consistency on Granularity

The SART measures ratio of change per time unit. Therefore, the monthly trend should be approximately four times the weekly trend and thirty times the daily trend. These correspondences are shown in Figure 2. Firstly, it is important to notice the substantial variation in the distribution of SART distances, which is one order of magnitude larger for ARIMA. Consequently, ARIMA has not been further considered for trend estimation. For the sake of clarity, the distribution of SART distances for OLS and WLS have been represented separately (see right graph in Figure 2). WLS presents the best performance in terms of granularity and the disparity with OLS is particularly visible for those scenarios in which independent time periods are used (1M_1_ and 7D_1_). All the distances estimated by WLS were significantly smaller (*p* < 0.001) than those obtained using OLS and ARIMA.

### 3.2. AMR Surveillance: Case Studies

The most commonly requested sample types were urine (30%), wound (26%) and blood (6%) with the majority of tests corresponding to a reduced set of pathogens which are a common cause of infection. The most representative pathogens were *Escherichia coli* (51% in urine cultures and 26% in blood cultures) and *Staphylococcus aureus* (49% in wound cultures). As such, to provide a detailed insight and validate the estimated resistance rates and trends, three case studies are presented below: (i) *Escherichia coli* in blood samples; (ii) *Escherichia coli* in urine samples; and (iii) *Staphylococcus aureus* in wound samples.

**Figure 3:**
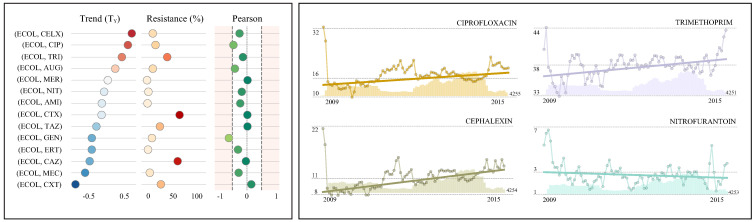
Case study I: *E. coli* in urine samples. The table presents the resistance index, resistance trend (monthly and yearly), the pearson correlation coefficient, the number of isolates and external resources for validation for each antimicrobial. In addition, the main metrics (**left**) and four examples of resistance time series (**right**) are graphically represented to facilitate comparison.

**Figure 4:**
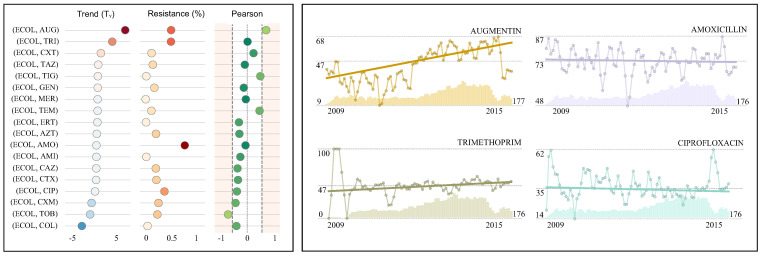
Case study II: *E. coli* in blood samples. The table presents the resistance index, resistance trend (monthly and yearly), the pearson correlation coefficient, the number of isolates and external resources for validation for each antimicrobial. In addition, the main metrics (**left**) and four examples of resistance time series (**right**) are graphically represented to facilitate comparison.

**Figure 5:**
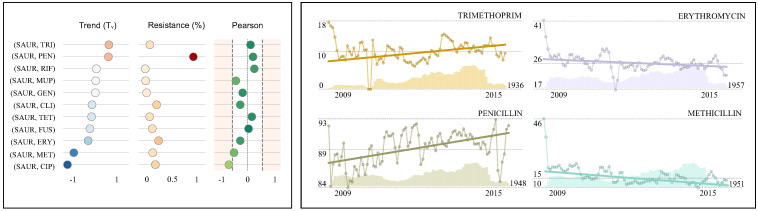
Case study III: *S.aureus* in wound samples. The table presents the resistance index, resistance trend (monthly and yearly), the Pearson correlation coefficient, the number of isolates and external resources for validation for each antimicrobial. In addition, the main metrics (**left**) and four examples of resistance time series (**right**) are graphically represented to facilitate comparison.

The presented case studies (see Table 2, Table 3 and Table 4, Figure 3, Figure 4 and Figure 5) report the resistance rates and resistance trends (monthly and yearly) with the corresponding confidence intervals for various antimicrobials. These results are supported with references to the existing literature. The last two columns present the Pearson correlation coefficient and the total number of isolates. For the sake of clarity, the resistance rate, the resistance trend and the Pearson correlation coefficient have been displayed graphically. In addition, a number of resistance time series have been represented graphically (see Table 2, Table 3 and Table 4, Figure 3, Figure 4 and Figure 5) including the number of susceptibility tests (bars), the corresponding resistance index (circle) and the estimated linear trend (overlay straight line).

## 4. Discussion

The process to generate a resistance time series signal from susceptibility data is defined by two parameters: shift and period. Regardless of the value of these parameters, the estimated trends should be independent of the granularity (shift) and show a consistent change when time spans overlap (period). OLS is perhaps the most popular method for trend estimation and has shown consistent results in our study. However, it is known to be greatly affected by outliers. To palliate this effect, WLS has been considered to reduce the contribution of outliers by considering the number of susceptibility tests available. While ARIMA is a very popular suite of models which has proven to be robust in short-term forecasting, it has two main limitations: (i)requires stationary time series and (ii) parameter tuning is not straightforward. Altogether, WLS was selected as the preferred method for trend estimation since it was robust against changes in the granularity of the time series and presented the best performance. In addition, it is easy to comprehend and use increasing its practicability and implementation in other institutions.

### 4.1. Case Study I: *Escherichia coli* in Urine Samples

*Escherichia coli* (ECOL) is a gram negative bacteria and most strains are harmless, being part of the normal flora of the gut. However, virulent strains can cause gastroenteritis, urinary tract infection, meningitis and Crohn’s disease. It is the most widely studied pathogen since it is easy to reproduce under favourable conditions. *E. coli* is responsible for more than 85% of all urinary tract infections. There is an alarming resistance to cefotaxime (60.8%), ceftazidime (57.3%) and trimethoprim (37.8%) with equivalent results in other studies (see table in Figure 3). Furthermore, there has been a noticeable increase in resistance to cephalexin (0.7%), ciprofloxacin (0.6%) and trimethoprim (0.4%). On the other side, resistance to Augmentin (0.2%) is positive yet not significant since the confidence intervals contain 0. Nitrofurantoin is commonly identified as one of the most active agents to treat *E. coli* with resistance rates within the range 3.7–6% in 2003–2008 [39,42], further stabilized to 3% in 2015–2017 [5,41]. These rates harmonize with those presented in the corresponding resistance time series (see time series graph for nitrofurantoin in Figure 3) and the estimated marginally decreasing trend (−0.01%). While the resistance rate to nitrofurantoin (2.7%) is low, there are antimicrobials with even lower rates such as ertapenem (2.0%) or amikacin (1.1%). Furthermore, carbapenems show negligible resistance rates; meropenem (0.2%) and imipenem (0.2%).

### 4.2. Case Study II: *Escherichia coli* in Blood Samples

The national mandatory surveillance program has reported a consistent rise in the incidences of *E. coli* bacteremia in England [5]. Furthermore, the majority of antimicrobials under surveillance also presented an increase in resistance rates over the last years (see Figure 4). Such rising resistance rates are particularly significant for Augmentin (4.3%) and trimethoprim (2.3%). Note that, although the resistance trend for Augmentin is significant, the pearson coefficient indicates a high correlation between the number of records and the estimated resistance index. The introduction of MALDI-TOF mass spectrometry in 2011 might have caused this effect (see time series graph for Augmentin in Figure 4). The high proportion of resistant isolates presented for these two antimicrobials (47.5% and 47.2% respectively) is only overpassed by amoxicillin (72.7%). Thus, there should be concerns on the use of these antimicrobials in clinical practice. Ciprofloxacin presents the fourth highest proportion of resistant isolates (35.2%), which has been slightly decreasing in recent years [27]. On the contrary, surveillance in carbapenems shows negligible resistance rates which have remained constant over the years [5]. For instance, meropenem and ertapenem resistance rates (0.5% and 1.2%) and trends (0.0% and −0.1%) are shown in Figure 4.

### 4.3. Case Study III: *Staphylococcus aureus* in Wound Samples

*Staphylococcus aureus* (SAUR) is a Gram-positive bacteria typically found in the respiratory tract and the skin. It is a leading cause of bloodstream infections [46,47], generally associated with breakages in the skin due to surgery, injury or use of intra-vascular devices such as catheters. Therefore, it is frequently acquired in hospitals [48]. Penicillin-resistant isolates were recognised in 1942 [49] reaching a proportion of 80% by late 1960s. Nowadays, the resistance rate to penicillin (89.4%) is the highest and has shown an increasing trend (0.6%) over the last years. There was also an emergence of methicillin-resistant *Staphylococcus aureus* (MRSA). It was first reported in a British hospital and became a worldwide problem in clinical medicine [27] with a peak of 43% in 2001 [50]. The Department of Health in England made reduction in rates of MRSA a priority with improvement of surveillance as one of their first actions. This led to a decrease in the number of resistant cases reported [51]. Such decrease continued in 2012–2015 [41,52] and coincides with the negative trend (−1.1%) presented in Figure 5 and the corresponding time series graph. Nowadays, approximately 15.3% of isolates are methicillin-resistant [41]. Also, there should be concerns on antimicrobials such as erythromycin (26.0%) and clindamycin (22.4%) with higher resistance rates and no further evidence of improvement. Moreover, while resistance rates to trimethoprim are not very high (10.1%), it has presented a noticeable rise (0.6%) in the last years. Since trimethoprim is clinically valuable to treat skin and soft tissue infections caused by MRSA, such rise constitutes a major threat. On the other side, rifampicin-containing treatments are known to improve outcomes in Staphylococcal wound infections presenting the lowest resistance rate (1.7%) and a constant trend (0.0%) [45].

### 4.4. Susceptibility Testing: Behaviour and Guidelines

The information extracted from antimicrobial surveillance is valuable to guide and support antimicrobial therapy selection. However, the reliability of this information highly depends on the number of observations available which might vary considerably (see isolates in Figure 3). For instance, a large proportion of combinations had an insufficient number of susceptibility tests to perform resistance trend estimation. The disparity among the pathogens tested is induced by the hospital occurrence rate; leading to higher number of tests for pathogens which are a common cause of infection in the population. On the other side, laboratory guidelines promote susceptibility testing for a limited range of antimicrobial agents that, based on pharmacological and empirical knowledge, may potentially be able to inhibit or kill the pathogen. This selection, generally based on national laboratory guidelines, causes the corresponding disparity among antimicrobials. Note that these guidelines might not be appropriate for the needs of each hospital. Thus, developing effective communication strategies, such as the resistance summaries presented in this work, could provide the necessary knowledge to revise and potentially tailor them accordingly.

### 4.5. Advantages of Overlapping Time Intervals in Surveillance

Antimicrobial surveillance is performed at different levels (e.g., local or national) and it is greatly affected by the size of the dataset considered. Versatile yet efficient analytic methods are required in those scenarios where data access or availability is restricted, such as clinical research. The main advantages of overlapping time intervals are: (i) it is a flexible approach which enables to adjust granularity and accuracy (ii) resulting time series are visually more legible and insightful (iii) enables the study of short-time variations in contrast to current mechanisms using sparse data points (years apart) and (iv) the outcomes are more consistent. On the other side, it might originate certain relationship between consecutive observations as data is partially shared. Overall, this step is optional and might be particularly useful in scenarios where data is limited but decent levels of granularity are still required.

### 4.6. The Importance of Surveillance Data

Despite global antimicrobial surveillance becoming a priority in recent years, homogeneity of antimicrobial policies does still produce different antimicrobial resistance outcomes [8]. For instance, it is widely documented that resistance rates are considerably higher in London than in the rest of UK, emphasizing the significance of local AMR surveillance. Health care organizations benefit from data on rates of antimicrobial resistance in many ways: (i) contributes to the evidence base used for formulation of national treatment guidelines (ii) can be used to assess the effectiveness and impact of interventions and (iii) has a key role in detecting the emergence and spread of previously uncommon or completely novel types of resistance. Furthermore, AMR surveillance plays a major role in patient management by providing data that influences clinical decision-making [9]. Since it guides antimicrobial selection for empirical treatment it is crucial at point of care. For such reason, this information will be integrated in Enhanced Personalized and Integrated Care for Infection Management at Point of Care (EPIC IMPOC), a modular intelligent decision support system which aims to assist clinicians at the different stages of the infection management pathway [53,54,55,56,57].

### 4.7. Limitations

The data considered in this study were not collected purposely. Therefore, it could be influenced by external factors such as changes in susceptibility testing policies (e.g., MIC breakpoints), technology or outbreaks. For instance, the introduction of MALDI-TOF mass spectrometry in 2011 might have affected the number of microbiology tests requested by clinicians. However, while testing relies on hospital policies, suspicion of infection was assumed for all microbiology tests requested.

## 5. Conclusions

Surveillance is the cornerstone for assessing the burden of antimicrobial resistance and strengthens knowledge for action in support of stewardship program strategies by improving existing guidelines. The efficient use of susceptibility data provided by the overlapping time spans drops the dependence between the granularity and accuracy of traditional surveillance systems. The robustness of weighted least squares regression facilitates resistance trend estimation and could be used to enhance existing surveillance systems which exclusively focus on resistant rates. Furthermore, there is an opportunity to investigate seasonal or other cyclic variations. Automating and facilitating access to surveillance reports through clinical decision support systems would enhance resistance awareness among clinicians and possibly have an impact on antimicrobial prescription practices.

## Figures and Tables

**Table 1:** Description of strategies to generate resistance time series.

**Independent time intervals**				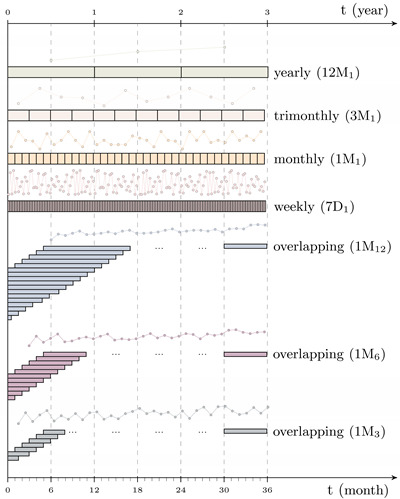
This is the traditional method used in antimicrobial surveillance systems where the time spans considered are independent; that is, they do not overlap (e.g., month or year).				
**Overlapping time intervals**				
This method is defined as a fixed region which is moved across time to compute consecutive resistance indexes. It is described by two parameters; the length of the region (period) and the distance between consecutive windows (shift).				
**Notation**				
The notation to define the time series generation methodology use is shift_period_. Some examples are presented below.				
shift_period_	Shift	Period	Type	
1D_1_	Daily	previous day	I	
7D_1_	Weekly	previous week	I		
1M_1_	Monthly	previous month	I	
12M_1_	Yearly	previous year	I	
1M_12_	Monthly	previous year	O	
1M_6_	Monthly	previous 6 months	O	
1M_3_	Monthly	previous 3 months	O	

**Keys:***D* = day; *M* = month; *I* = independent time intervals; *O* = overlapping time intervals.

**Table 2:** AMR summary for *E. coli* in urine samples.

Antimicrobial	R(%) (95% CI)		References	T_M_(%) (95% CI)		References	T_Y_(%)		Pearson	Isolates
Cephalexin (CELX)	**11.1**	(10.9, 11.3)		**0.055**	(0.045, 0.065)		0.7	↑	−0.25	79,090
Ciprofloxacin (CIP)	**16.3**	(16.0, 16.5)	[39,40]	**0.046**	(0.031, 0.062)	[5,40]	0.6	↑	−0.46	79,239
Trimethoprim (TRI)	**37.8**	(37.4, 38.1)	[39,40,41,42]	**0.033**	(0.020, 0.046)	[40]	0.4	↑	−0.14	79,133
Augmentin (AUG)	**10.9**	(10.7, 11.2)		**0.018**	(−0.022, 0.059)		0.2	↔	−0.42	79,093
Meropenem (MER)	**0.2**	(0.1, 0.3)		**0.002**	(−0.002, 0.006)		0.0	↔	0.02	9875
Nitrofurantoin (NIT)	**2.7**	(2.6, 2.8)	[39,40,41,42]	**−0.006**	(−0.013, 0.001)		−0.1	↔	−0.18	79,108
Amikacin (AMI)	**1.1**	(0.9, 1.2)		**−0.011**	(−0.022, 0.000)		−0.1	↔	−0.23	9786
Cefotaxime (CTX)	**60.8**	(59.9, 61.8)		**−0.012**	(−0.083, 0.059)		−0.1	↔	0.01	9803
Tazocin (TAZ)	**24.2**	(23.3, 25.0)	[39]	**−0.023**	(−0.078, 0.032)		−0.3	↔	0.01	9878
Gentamicin (GEN)	**9.3**	(9.1, 9.5)	[42]	**−0.033**	(−0.061, −0.005)		−0.4	↓	−0.62	63,399
Ertapenem (ERT)	**2.0**	(1.7, 2.3)		**−0.033**	(−0.050, −0.017)		−0.4	↓	−0.31	8882
Ceftazidime (CAZ)	**57.3**	(53.3, 58.2)		**−0.038**	(−0.113, 0.037)		−0.5	↔	−0.04	9810
Mecillinam (MEC)	**5.4**	(4.9, 5.8)		**−0.048**	(−0.071, −0.024)		−0.6	↓	−0.29	9083
Cefoxitin (CXT)	**26.0**	(25.1, 26.8)		**−0.069**	(−0.123, −0.016)		−0.8	↓	0.15	9798

**Keys:** CI = confidence interval; R = resistance; T_M_ = monthly trend; T_Y_ = yearly trend; ↑ = significant increase; ↓ = significant decrease. **Significance:** A trend is significant if the CI does not include 0.

**Table 3:** AMR summary for *E. coli* in blood samples.

Antimicrobial	R(%) (95% CI)		References	T_M_(%) (95% CI)		References	T_Y_(%)		Pearson	Isolates
Augmentin (AUG)	**47.5**	(45.8-49.2)	[5,41]	**0.359**	(0.249, 0.470)	[41]	4.3	↓	0.64	3317
Trimethoprim (TRI)	**47.2**	(45.4–49.1)		**0.190**	(0.079, 0.301)		2.3	↓	0.01	2774
Cefoxitin (CXT)	**11.5**	(10.4–12.6)		**0.041**	(−0.006, 0.089)		0.5	↔	0.22	3316
Tazocin (TAZ)	**13.7**	(12.6–14.9)	[5,41,43]	**0.006**	(−0.040, 0.052)	[5,41,43]	0.1	↔	−0.08	3321
Tigecycline (TIG)	**1.7**	(1.2–2.2)		**0.002**	(−0.026, 0.030)		0.0	↔	0.45	2734
Gentamicin (GEN)	**16.6**	(15.3–17.8)	[5,44]	**0.000**	(−0.044, 0.045)	[5,44]	0.0	↔	−0.12	3322
Meropenem (MER)	**0.5**	(0.3–0.8)	[5,41,43,44]	**−0.001**	(−0.020, 0.018)	[5,41,43,44]	0.0	↔	−0.05	3280
Temocillin (TEM)	**11.1**	(10.0–12.2)		**−0.002**	(−0.086, 0.082)		0.0	↔	0.42	3044
Ertapenem (ERT)	**1.2**	(0.8–1.6)		**−0.005**	(−0.025, 0.016)		−0.1	↔	−0.28	2992
Aztreonam (AZT)	**19.6**	(18.1–21.0)		**−0.012**	(−0.077, 0.052)		−0.1	↔	−0.26	2925
Amoxicillin (AMO)	**72.7**	(71.2–74.2)		**−0.017**	(−0.085, 0.051)		−0.2	↔	−0.06	3319
Amikacin (AMI)	**1.6**	(1.2–2.1)		**−0.018**	(−0.041, 0.006)		−0.2	↔	−0.23	3044
Ceftazidime (CAZ)	**19.3**	(17.9–20.6)	[27,44]	**−0.019**	(−0.065, 0.027)	[27]	−0.2	↔	−0.33	3323
Cefotaxime (CTX)	**20.3**	(18.9–21.7)	[27,44]	**−0.021**	(−0.070, 0.027)	[27]	−0.3	↔	−0.31	3201
Ciprofloxacin (CIP)	**35.2**	(33.6–36.8)	[44]	**−0.035**	(−0.017, 0.037)		−0.4	↔	−0.35	3320
Cefuroxime (CXM)	**24.2**	(22.8–25.7)		**−0.080**	(−0.137, −0.024)		−1.0	↓	−0.39	3320
Tobramycin (TOB)	**22.1**	(20.6–23.6)		**−0.099**	(−0.188, -0.010)		−1.2	↓	−0.65	2832
Colistin (COL)	**4.0**	(3.3–4.8)		**−0.208**	(−0.274, −0.141)		−2.5	↓	−0.37	2606

**Keys:** CI = confidence interval; R = resistance; T_M_ = monthly trend; T_Y_ = yearly trend; ↑ = significant increase; ↓ = significant decrease. **Significance:** A trend is significant if the CI does not include 0.

**Table 4:** AMR summary for *S. aureus* in wound samples.

Antimicrobial	R(%) (95% CI)		References	T_M_(%) (95% CI)		References	T_Y_(%)		Pearson	Isolates
Trimethoprim (TRI)	**10.1**	(9.8, 10.4)		**0.052**	(0.026, 0.077)		0.6	↓	0.10	33,525
Penicillin (PEN)	**89.4**	(89.1, 89.7)		**0.050**	(0.034, 0.065)		0.6	↓	0.19	39,901
Rifampicin (RIF)	**1.7**	(1.5, 1.8)	[45]	**0.001**	(−0.015, 0.017)	[45]	0.0	↔	0.23	35,141
Mupirocin (MUP)	**2.5**	(2.3, 2.6)		**−0.001**	(−0.020, 0.017)		0.0	↔	−0.39	33,716
Gentamicin (GEN)	**4.1**	(3.9, 4.3)		**−0.003**	(−0.023, 0.018)		0.0	↔	−0.16	35,255
Clindamycin (CLI)	**22.4**	(22.0, 22.8)		**−0.016**	(−0.034, 0.001)		−0.2	↔	−0.24	39,962
Tetracycline (TET)	**9.7**	(9.4, 10.0)		**−0.018**	(−0.041, 0.004)		−0.2	↔	0.15	35,429
Fusidic acid (FUS)	**14.5**	(14.2, 14.9)		**−0.025**	(−0.044, −0.006)		−0.3	↓	0.04	39,918
Erythromicin (ERY)	**26.0**	(25.6, 26.5)		**−0.032**	(−0.049, −0.015)		−0.4	↓	−0.24	39,971
Meticillin (MET)	**15.3**	(14.9, 15.7)	[41]	**−0.090**	(−0.113, −0.068)	[41]	−1.1	↓	−0.45	39,950
Ciprofloxacin (CIP)	**20.1**	(19.7, 20.5)		**−0.116**	(−0.156, −0.075)		−1.4	↓	−0.62	35,227

**Keys:** CI = confidence interval; R = resistance; T_M_ = monthly trend; T_Y_ = yearly trend; ↑ = significant increase; ↓ = significant decrease. **Significance:** A trend is significant if the CI does not include 0.

## Data Availability

The processed datasets analysed during the current study are available from the corresponding author (B.H.) on reasonable request, as long as this meets local ethics and research governance criteria.

## Abbreviations

The following abbreviations are used in this manuscript:

ADF	Augmented Dickey-Fuller
AMR	Antimicrobial Resistance
AMS	Antimicrobial Stewardship
AR	Autoregressive
ARIMA	Autoregressive Integrated Moving Average
ARMA	Autoregressive Moving-Average
BIC	Bayesian Information Criterion
CLSI	Clinical and Laboratory Standards Institute
ECOL	*Escherichia coli*
EUCAST	European Committee on Antimicrobial Susceptibility Testing
KPSS	Kwiatkowski-Phillips-Schmidt-Shin
MA	Moving Average
MALDI-TOF	Matrix Assisted Laser Desorption/Ionization-Time Of Flight
MARI	Multiple Antimicrobial Resistance Index
MIC	Minimum Inhibitory Concentration
MRSA	Methicillin Resistant *Staphylococcus aureus*
NHS	National Health Service
OLS	Ordinary Least Squares
SARI	Single Antimicrobial Resistance Index
SARIMA	Seasonal Autoregressive Integrated Moving Average
SART	Single Antimicrobial Resistance Trend
SAUR	*Staphylococcus aureus*
VARIMA	Vector Autoregressive Integrated Moving Average
WLS	Weighted Least Squares
